# Street Collections

**Published:** 1892-07-30

**Authors:** 


### Passing Topics.

### STREET COLLECTIONS.

This year something more than the customary amount of public grumbling has taken place in respect of the solicitations of the Hospital Saturday Fund street collectors, with a decided tendency on the part of the Press to sympathise with the complainants, one journal going so far as to say that the begging was distinctly of the "stand and deliver" order. We think, therefore, it would be to the advantage of the fund if the authorities were to consider how the street collection, if retained at all, can be made more popular, which at the same time means more effective, than it now is.

To pretend that, as at present conducted, it proves anything but public indifference would be absurd, in the face of the figures. According to the published returns, twelve hundred boxes were placed in position. These must have been attended by at least double the number of collectors, and in appraising the result we have to bear in mind the fact that all these workers were more or less fair and fascinating. He would indeed be a bold man and a foolish who, in the presence of twenty-four hundred ladies so pertinacious, would seek to apportion a greater power of allurement to the fund than to its advocates: in other words, it would be no less indiscreet than untrue to hint that the same champions would not be capable of accomplishing quite as great things in any other cause.

The twenty-four hundred ladies collected between them £1,902, after a long, and as it must have appeared to most of them, a tedious day's work, happily not rendered worse this year by inclemencies of weather. If we take the gold (£168) as representing two givers for each sovereign, the silver (£955) as contributed in sixpences, and the bronze (£750) in pence, we have an array of some 200,000 donors, or about one in twelve of the pedestrians who passed one or more of the boxes, allowing for infants without pockets, the penniless, and that portion of the population which remained indoors throughout the day. Of the donors less than 20,000 gave more than one penny apiece.

It would be easy to compare the usual cheap homily concerning the meanness of the multitude; but what more concerns the present purpose is to remark upon the amount of unrewarded labour undertaken by the devoted twenty-four hundred. We have assumed that the pockets of about one-half the actual population, or some two and a half millions in all, were assailable by the fair forces, and so admirably placed were their pickets, that every approach was under observation, and it was nothing unusual for the wayfarer to be called upon to defend both flanks at once. Judging by our own experience, it would be safe to multiply the two and a-half millions of pockets by fifty, in order to arrive at an approximate estimate of the total number of assaults delivered, and when these figures are worked out we find that the average result of each effort was, in a sense, incalculable.

Looked at in this way, we see little to commend a street collection, even for so meritorious an object as the maintenance of the hospitals. But there is more to urge. Expenses must be deducted, of course, but they are as nothing compared with the more formidable item of losses. Street collections, like street parades, appear to many quiet people nothing less than a nuisance. There is as good reason for believing that many are offended by these methods as that the Ladies' Legion of the Saturday Fund is capable of accomplishing better things with better opportunities. We flatter ourselves that if we had 2,400 zealous ladies at command, and a good cause to serve, we should set them a task very different from that which appears to content the modest ambitions of the Saturday Fund authorities. Agricola.

